# Digital CRISPR-Powered Biosensor Concept without Target Amplification Using Single-Impact Electrochemistry

**DOI:** 10.1021/acssensors.4c02060

**Published:** 2024-10-22

**Authors:** Sebastian Freko, Marta Nikić, Dirk Mayer, Lennart J. K. Weiß, Friedrich C. Simmel, Bernhard Wolfrum

**Affiliations:** † Neuroelectronics, Munich Institute of Biomedical Engineering, Department of Electrical Engineering, School of Computation, Information and Technology, Technical University of Munich, 85748 Garching, Germany; ‡ Institute of Biological Information Processing, Bioelectronics (IBI-3), Forschungszentrum Jülich, 52425 Jülich, Germany; § Department of Bioscience, TUM School of Natural Sciences, Technical University of Munich, 85748 Garching, Germany

**Keywords:** single-impact electrochemistry, silver nanoparticles, freezing functionalization, CRISPR-based diagnostics, amplification-free digital sensing

## Abstract

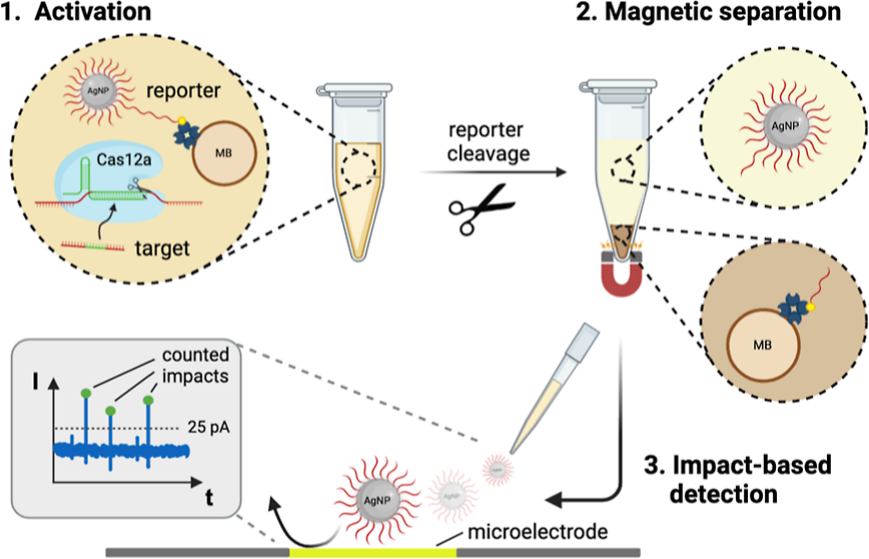

The rapid and reliable detection and quantification of nucleic acids is crucial for various applications, including infectious disease and cancer diagnostics. While conventional methods, such as the quantitative polymerase chain reaction are widely used, they are limited to the laboratory environment due to their complexity and the requirement for sophisticated equipment. In this study, we present a novel amplification-free digital sensing strategy by combining the collateral cleavage activity of the Cas12a enzyme with single-impact electrochemistry. In doing so, we modified silver nanoparticles using a straightforward temperature-assisted cofunctionalization process to subsequently detect the collision events of particles released by the activated Cas12a as distinct current spikes on a microelectrode array. The functionalization resulted in stable DNA-AgNP conjugates, making them suitable for numerous biosensor applications. Thus, our study demonstrates the potential of clustered regularly interspaced short palindromic repeats-based diagnostics combined with impact-based digital sensing for a rapid and amplification-free quantification of nucleic acids.

Over the past decade, rapid and inexpensive detection of nucleic acids at the point-of-care (POC) has become of tremendous interest to public health, not only due to the Coronavirus disease 2019 pandemic, but also because of the potential use of nucleic acid detection and quantification as biomarkers, e.g., in cancer diagnostics.^1−4^ Conventional methods such as quantitative polymerase chain reaction (qPCR) and the reverse transcription quantitative real-time PCR (RT-qPCR) are time-consuming, expensive, and require sophisticated laboratory equipment and trained operators, limiting their widespread use to well-equipped laboratories.^5,6^ Although isothermal alternatives such as loop-mediated isothermal amplification (LAMP) or recombinase polymerase amplification have been developed, still the preamplification increases the detection time and can lead to amplification-related false-negative or -positive results.^7,8^ In addition to amplification-based diagnostics, cytological methods are complementarily used in screening for virus-associated cancers, such as cervical cancer. However, the lower sensitivity and higher costs lead, for example, to screening approaches based on testing for human papillomavirus DNA in cervical cancer.^9−11^ An emerging strategy for the detection and quantification of nucleic acids at the POC is the clustered regularly interspaced short palindromic repeats (CRISPR)-based diagnostic, which relies on the specificity, programmability, and ease of use of the CRISPR technology.^6^ CRISPR/Cas (CRISPR-associated) systems are RNA-mediated adaptive immune systems of bacteria and archaea.^12^ In 2012, the discovery of the Cas9 enzyme and its ability to introduce site-specific double-stranded breaks in DNA paved the way for programmable CRISPR/Cas9 systems for gene targeting and genome editing applications.^13^ More recently, other Cas enzymes, e.g., Cas12 and Cas13, have been discovered and utilized for detecting nucleic acids.^7,14,15^ In contrast to Cas9 systems, these types are characterized by a nonspecific collateral cleavage activity, also known as trans cleavage, after the recognition and cleavage of the target sequence. Since then, these systems have been applied in various biosensor concepts using different strategies, including fluorescent, colorimetric, and electrochemical readouts.^16−24^ However, most of the current methods must make trade-offs between sensitivity, response time, and ease of use. For example, some approaches provide only qualitative results (lateral flow strips), while others rely on preamplification or are not POC applicable since they need bulky readout devices. Over the past decade, a novel concept of “digital” sensing using single-impact electrochemistry has been employed.^25−35^ Thereby, redox-active entities such as silver nanoparticles (AgNPs) collide with a microelectrode, causing a distinct current spike (“1”) compared to the background noise (“0”). By simply counting the impacts during a defined measurement time, the concentration of the target species can be correlated to the mean impact frequency.^36−38^ Since impact-based digital sensing has a theoretical limit of detection (LOD) of a single entity, it could be the basis for next-generation amplification-free POC sensors. For instance, the concept has been implemented in a microfluidic paper-based analytical device and a lateral flow competitive binding assay.^38,39^ However, one major challenge of translating this readout toward relevant targets and applications is the design of colloidally stable nanoparticle labels, since the particles are typically sensitive to various parameters in the analyte solution, e.g., elevated salt concentrations.^40^

In this work, we combined the collateral cleavage activity of the Cas12a enzyme with a digital sensing strategy based on the oxidation events of DNA-coated AgNPs on a microelectrode array (MEA), as can be seen in Figure 1. We synthesized the DNA-AgNP conjugates using a straightforward temperature-assisted cofunctionalization procedure and immobilized them on streptavidin-coated magnetic beads (MBs).^41,42^ Upon activating the Cas12a using the target sequence, in our case HPV-16, the DNA-AgNP-MB reporter complex is cleaved, and the released particles can be detected by their collisions on the MEA. Thus, we extended the field of CRISPR-based diagnostics with an amplification-free digital sensor concept for potential POC applications.

**Figure 1:**
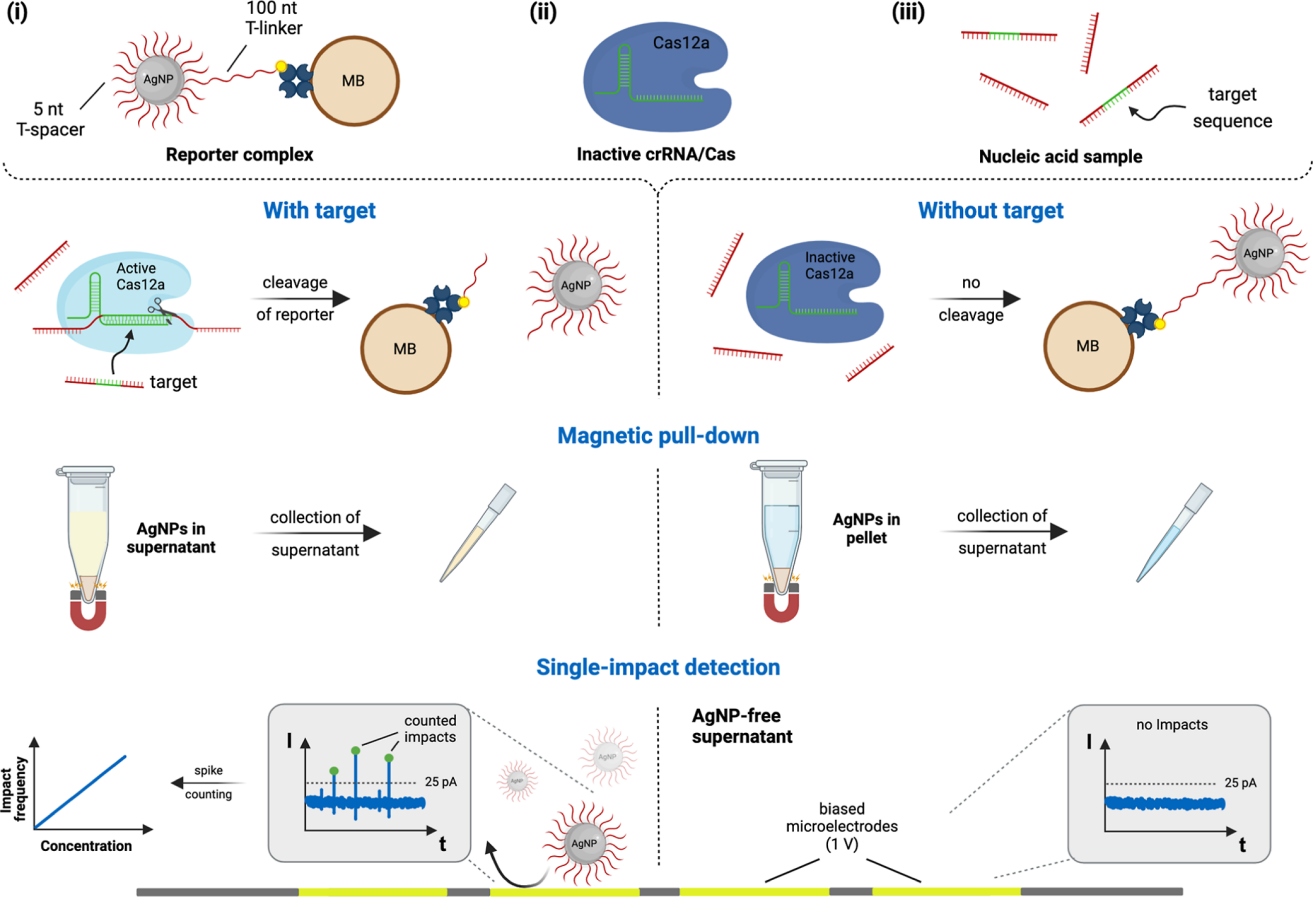
Amplification-free digital CRISPR-powered biosensor concept based on single-impact electrochemistry. The assay includes (i) a reporter complex consisting of DNA-AgNPs linked to MBs, (ii) the crRNA/Cas12a complex, and (iii) a sample potentially containing the target sequence. In the presence of the target, the collateral cleavage activity of Cas12a leads to the cleavage of the DNA-AgNP-MB reporter complex. After magnetic separation, released DNA-AgNPs are applied on a biased MEA, and particle oxidation occurs. Current peaks exceeding a threshold of 25 pA are counted as valid AgNP impacts. In contrast, if the target sequence is missing, Cas12 stays inactive and the reporter complex remains intact—no particles are released, and therefore, no impacts are visible. This figure was created with BioRender.com.

## Materials and Methods

### Materials

20 nm-sized AgNPs (citrate-capped, 0.02 mg/mL in aqueous solution), nitric acid (HNO_3_), sulfuric acid (H_2_SO_4_), TE buffer [10 mM Tris–HCl (pH 8,0) 0,1 mM ethylenediaminetetraacetic acid (EDTA)], 4-(2-hydroxyethyl)-1-piperazineethanesulfonic acid (HEPES), Tris(2-carboxyethyl)phosphin (TCEP), magnesium chloride (MgCl_2_), potassium chloride (KCl), potassium hydroxide (KOH), hydrogen peroxide (H_2_O_2_), and Tween-20 were purchased from Sigma-Aldrich (St. Louis, US). Tris(hydroxymethyl)aminomethane hydrochloride (Tris-HCl), EDTA was obtained from Carl Roth (Karlsruhe, Germany). Ammonium hydroxide (NH_4_OH) was bought from VWR Chemicals (Fontenay-sous-Bois, France). Alt-R LbCas12a *Ultra* and all oligonucleotide sequences, including the crRNA, were obtained from Integrated DNA Technologies (Coralville, US) and are listed in Table S1. 10× NEBuffer r2.1 (reaction buffer) was bought from New England Biolabs (Ipswich, US). Dynabeads MyOne Streptavidin C1 were purchased from Thermo Fisher Scientific (Waltham, US). Sodium chloride (NaCl) was obtained from neoLab Migge (Heidelberg, Germany). Nuclease-free water was bought from Qiagen (Hilden, Germany). Deionized water (conductivity 0.054 μS/cm) to prepare all solutions was taken from a Berry Pure purification system (Berrytec, Harthausen, Germany). The material costs of the implemented assay are summarized in Table S2.

### Temperature-Assisted Functionalization of AgNPs

**Figure 2:**
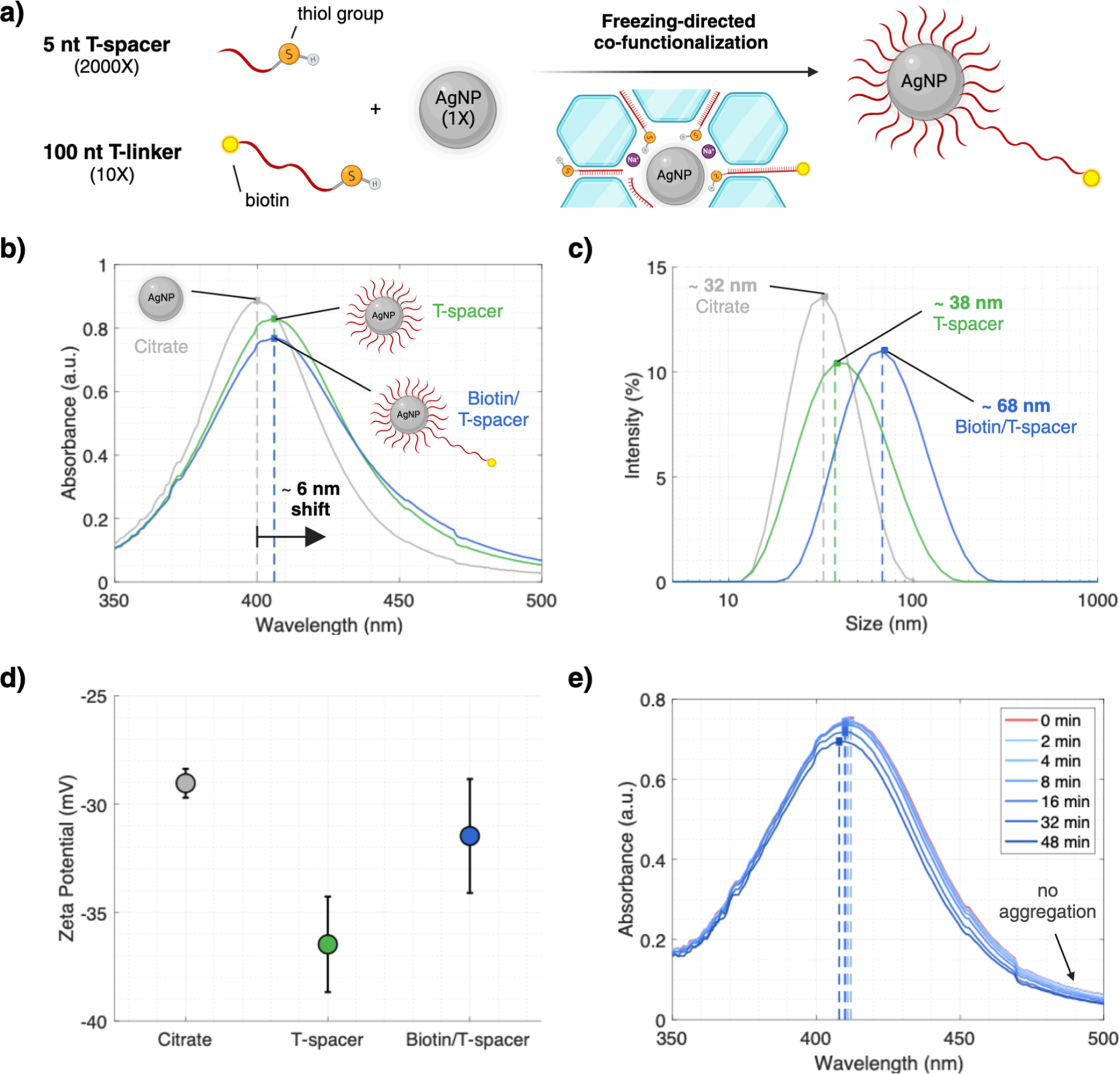
Freezing-directed cofunctionalization of AgNPs leads to stable DNA-AgNP conjugates. (a) Biotinylated T-linker (100 nt) and T-spacer (5 nt) are mixed with 20 nm AgNP using a molar mixing ratio of 10:2000:1. Upon freezing, the DNA is aligned and locally concentrated due to the formation of ice crystals–covalent binding takes place via the thiol group. The successful DNA attachment is indicated by (b) a redshift of the localized surface plasmon resonance (LSPR) peak (UV/vis), (c) a larger hydrodynamic diameter [dynamic light scattering (DLS)], and (d) a change in the ζ-potential. Error bars represent the mean ± standard deviation, where *n* = 3. (e) Biotin/T-spacer-AgNPs show no sign of aggregation for <45 min in 500 mM NaCl. This figure was created with BioRender.com.

A freezing-directed method to functionalize gold nanoparticles (AuNPs) with DNA was suggested by Liu and Liu, which we adopted for AgNPs.^41,42^ Similar to their protocol, we synthesized DNA-AgNP conjugates, as schematically shown in Figure 2a. We used AgNPs instead of AuNPs or platin nanoparticles (PtNPs) due to their lower oxidation potential that allows the direct observation of nanoparticle impacts at Pt microelectrode arrays. 20 nm AgNPs were functionalized with short T-spacers (5×T) to increase colloidal stability and long biotinylated T-linkers (100×T) to subsequently immobilize the particles to streptavidin-coated MBs. First, the protection group of the thiol-modified DNAs was removed via a reduction step by incubating 100 μM DNA (stored in TE buffer) with 100× excess of TCEP for 2 h at room temperature. Prior to mixing the AgNPs with DNA, the particles were concentrated to 5× (∼3.75 nM) by centrifugation at 16.000 rpm for 35 min. Activated biotinylated DNA (1 μM) and T-spacer (50 μM) were then coincubated with the AgNPs at a molar ratio of 10:2000:1 at −20 °C until the mixture was completely frozen. Subsequently, the particles were thawed at room temperature. After completing the freeze–thaw cycle, the suspension was washed with 900 μL HEPES (5 mM) by centrifuging three times at 16.000 rpm for 35 min each to remove unbound DNA.

### Characterization of DNA-AgNP Conjugates

The LSPR of the functionalized DNA-AgNPs was characterized using UV/vis spectroscopy (Analytik Jena Specord 200; Jenoptik; Jena, Germany). In addition, the hydrodynamic diameter and the ζ-potential were measured using a Zetasizer Nano ZS (Malvern Panalytical, Malvern, U.K.). While the ζ-potential measurements were carried out in a folded capillary zeta cell, the DLS and UV/vis spectra were measured in disposable polystyrol semimicro cuvettes (VWR International; Radnor, US). The same sample was used for all methods −50 μL of the citrate-capped AgNP were diluted in 750 μL dH_2_O, and 10 μL of DNA-AgNPs (5×) were added in 790 μL dH_2_O, respectively. Furthermore, the stability of the functionalized AgNPs was assessed by recording the UV/vis spectra in 500 mM NaCl at different points in time (2–48 min). Finally, the number of bound T-spacers per AgNP (functionalized with T-spacers only) was estimated. The particles were dissolved using a 14% NH_4_OH/2.5% H_2_O_2_ solution at a mixing ratio of 5:1. After dissolution, a small Pt wire (∼1 cm, curled) was added overnight to remove H_2_O_2_. The DNA concentration in the solution was measured using NanoDrop 8000 (Thermo Fisher Scientific; Waltham, US). Dissolved citrate AgNPs were used as blank.

### Preparation of DNA-AgNP-MB Reporter Complex

First, the MBs were washed once in a washing buffer (10 mM Tris-HCl pH = 7.5, 1 mM EDTA, 2 M NaCl) and then three times in binding buffer (10 mM Tris-HCl pH = 7.5, 1 mM EDTA). 0.1% Tween-20 was added to both buffers to reduce nonspecific binding. Afterward, the concentration of the MBs was adjusted to 5 mg/mL using the binding buffer. The DNA-AgNP-MB reporter complex was obtained by incubating the DNA-AgNP conjugates with the washed MBs at a volume ratio of 4:1 for 15 min at room temperature using gentle rotation in a composition of 2.5 mM HEPES and 100 mM NaCl. After coupling the DNA-AgNPs to the MBs via biotin/streptavidin, the reporter complex was washed with 1 mL of 5 mM HEPES using magnetic pull-down to remove unbound DNA-AgNP conjugates. The assembled DNA-AgNP-MB reporter complexes were stored in nuclease-free water at 4 °C until further usage.

### CRISPR-Powered Release and Magnetic Pull-Down

It is well-known that the trans cleavage activity highly depends on several factors, including temperature, ionic strength, and activator length. Therefore, the ribonucleoprotein (RNP) complexes were preassembled and activated according to the manufacturer’s instructions and literature.^15^ In brief, the RNP complexes were preassembled by incubating 200 nM Cas12a with 250 nM crRNA for 20 min at room temperature. Afterward RNP complexes were stored at 4 °C. The activation was done by incubation of 6 μL synthetic HPV-16 DNA sample (40 pM, 400 pM, and 4 nM) with 3 μL of the preassembled RNP complexes and 21 μL nuclease-free H_2_0 for 30 min at 37 °C. Finally, the reaction was initiated by mixing 6 μL of the activated RNP complexes (20 nM Cas12a: 25 nM crRNA) with 6 μL NEBuffer (10×) and 48 μL of DNA-AgNP-MB reporter complex. The reaction was incubated at 37 °C for 15 min and stopped by adding EDTA in 10-fold excess. Subsequently, the sample was placed in a magnetic rack to pull down the MBs. Depending on the HPV-16 concentration, DNA-AgNPs were released and quantified using UV/vis spectrometry and single-impact detection.

### UV/Vis Quantification of Released DNA-AgNPs

After the cleavage of the DNA-AgNP-MB reporter complex and the separation of the MBs and the DNA-AgNPs using magnetic pull-down, the released DNA-AgNPs were first quantified by recording the UV/vis spectra. For this experiment, the AgNP concentration was measured using the NanoDrop since only an analyte volume of 2 μL is required, and no further dilution of the sample is necessary. Maximum extinction values were extracted.

### Single-Impact Experiments and Evaluation

In addition to the UV/vis readout, we combined the collateral cleavage activity of Cas12a with a digital readout based on single-impact electrochemistry. The fabrication of the MEA chips used is described briefly. A metal stack consisting of subsequent layers of 20 nm Ti (bottom adhesion layer), 200 nm Pt (electrode), and 5 nm Ti (top adhesion layer) was deposited on a 500 μm-thick Borofloat wafer using electron beam evaporation. Standard photolithography was used to pattern a spin-coated double-layer resist (LOR 3b, Microchem, Newton, MA and AZ nLOF 2070, MicroChemicals, Ulm, Germany). The metal structures were passivated using a 40 nm layer of Ta_2_O_5_ and an 800 nm stack of five alternating layers of silicon oxide (200 nm) and silicon nitride (100 nm), starting with silicon oxide. Reactive ion etching was used to remove the passivation and the top Ti adhesion layer at the electrode openings/contact pads. The final MEA comprises 62 detection electrodes (8 μm diameter) and two large quasi-reference electrodes (100 μm diameter). A chemical cleaning procedure was applied before every experiment to obtain consistent impact results, using the same chip multiple times.^29^ In summary, the four-step cleaning consists of chronoamperometry in NH_4_OH (−2 V for 60 s), a short incubation in HNO_3_, electrochemical reduction in KOH (−1.5 V for 210 s), and finally, cyclic voltammetry in 0.2 M H_2_SO_4_ solution (within a potential range from −0.2 to 1.5 V at a scan rate of 500 mV/s over 21 cycles) to assess the state of the electrode. The entire protocol was performed prior to an experiment, while only the last two steps were used between two measurements of the same experiment. All electrochemical cleaning steps were conducted within a three-electrode configuration using a VSP-300 potentiostat (BioLogic Instruments, France) comprising an Ag/AgCl reference electrode (Dri-ref, World Precision Instruments) and a coiled platinum wire as the counter electrode. The detection experiments were carried out in a 2-electrode configuration using a custom-built 64-channel amplifier system (10 kHz sampling frequency per channel, 3.4 kHz bandwidth), shown in Figure S1. The detection conditions for the calibration curve (impact frequency as a function of HPV-16 concentration) were as follows: 100 μL of 400 mM KCl (final 200 mM), 20 μL of 500 mM KOH (final 50 mM), 30 μL deionized water, and 50 μL released DNA-AgNPs, resulting in a total volume of 200 μL. The obtained data was analyzed using a MATLAB code that includes the detrending of the current traces, a conservative current threshold of 25 pA to avoid misclassification of AgNP impacts, and a minimum interpeak distance of 10 ms.^43^ Unless otherwise stated, the ten best-performing channels are shown in all experiments, and outliers are removed by the 2σ-interval to account for manufacturing errors. As the same chip was used for each subset of measurements of an experiment, identified outlier channels were removed for all measurements of this experiment.

## Results and Discussion

### Characterization of DNA-AgNP Conjugates

After completing the freeze–thaw cycle and washing the particles, we assessed the functionalization outcome by considering the particles’ plasmonic behavior, their hydrodynamic diameters, and their ζ-potentials. Upon freezing, the local concentration of salt, AgNPs, and DNA increases in the gaps of the ice crystals.

Additionally, the DNA is aligned, stretched, and comes into proximity of the AgNP surface, allowing covalent bonding via the thiol group (see Figure 2a).^42^ The successful thiol bond formation was indicated by a redshift (∼6 nm) of the LSPR peak, as can be seen in Figure 2b.^44,45^ Furthermore, a larger hydrodynamic diameter and a change in the zeta potential confirmed the successful DNA attachment (see Figure 2c,d). Interestingly, the redshift of the LSPR peak was the same for AgNPs functionalized with T-spacers and AgNPs functionalized with long biotinylated T-linkers and T-spacers. A possible explanation for this is that the LSPR peak shift is dominated not only by the length of the ligands but also by the number of bonds formed.^46^ T-spacers were present in 200-fold excess and are generally relatively large (five nucleotides plus C3-spacer) compared to other ligands, such as short alkanethiols or 3-mercaptopropionic acid. Therefore, the shift is likely dominated by the large number of bound T-spacers since the evanescent field drops exponentially and is most sensitive to changes very close to the metal surface. On the other hand, the presence of the 20 times longer biotinylated T-linkers had a prominent effect on the hydrodynamic diameter due to a bulkier solvation shell (see Figure 2c). Finally, the UV/vis spectrum of the functionalized AgNPs was monitored for up to 48 min in a high-salt electrolyte of 500 mM NaCl, showing only minimal changes in the signal (see Figure 2e). The results indicate colloid stability due to electrostatic repulsion of the protective DNA shell. This suggests that the particle-conjugates are also suitable for our assay with detection conditions at lower salt concentrations of 200 mM KCl/50 mM KOH (∼3 min) or the cleavage conditions of the Cas12 enzyme (10 mM MgCl_2_ for 15 min). Their colloid stability in such high salt concentrations, a common challenge in utilizing metal nanoparticles,^40,47,48^ could make the reporter complex suitable for a broad range of biosensor applications.

### Characterization and Optimization of the Reporter Complex

**Figure 3:**
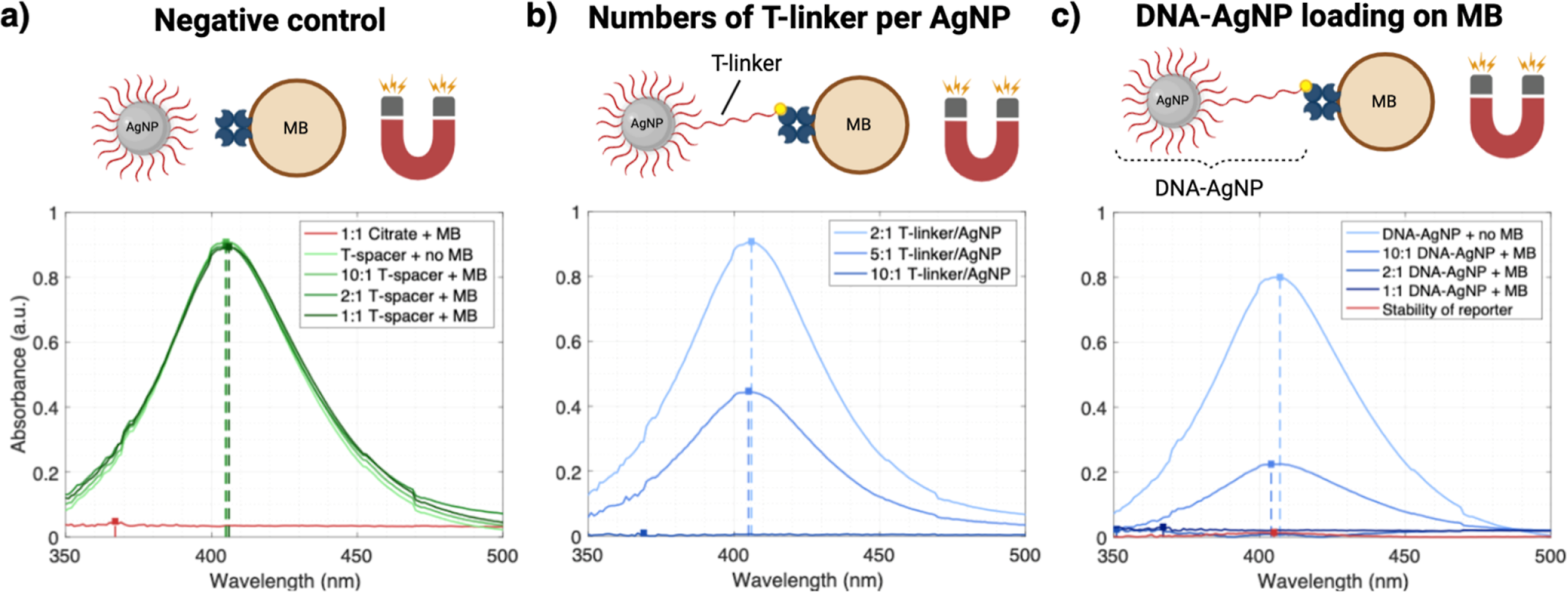
Characterization and optimization of the magnetic pull-down concept. (a) The T-spacer shell is required to prevent citrate-capped AgNPs from aggregation and adsorption to the MBs. The successful DNA attachment protects the AgNPs even at a T-spacer/MB volume ratio of 1:1—no sign of aggregation/adsorption is visible since the amplitude does not decrease. (b) Different biotinylated T-linker ratios per AgNP were tested, and the UV/vis spectra of the supernatant were measured. A ratio of 10:1 is required to ensure that almost every particle gets at least one T-linker and, therefore, is pulled down after binding to the MB. (c) The DNA-AgNP loading on the MBs was determined by incubating the functionalized particles with the MBs at different mixing ratios. In addition, the stability of the assembled reporter complex was tested for 1 week in deionized water in the fridge. This figure was created with BioRender.com.

In addition to the high stability attributed to the T-spacer shell, the DNA layer also reduces nonspecific adsorption to the MBs (see Figure 3a). For instance, citrate-capped particles adsorb to the MBs and aggregate in the binding buffer solution. Highlighting the importance of the T-spacer shell, we tuned the mixing ratio of the biotinylated T-linker to the AgNP. Since the particle corona has an essential influence on the redox activity of the particle, we aimed for as few long DNA strands per particle as possible. At the same time, each AgNP should at least get one T-linker to bind to an MB. We found that a 10:1 ratio of T-linker to AgNP sufficiently fulfilled this trade-off (see Figure 3b). Next, we determined the loading capacity of the MBs by increasing the MB concentration while keeping the DNA-AgNP concentration constant (see Figure 3c). The average number of AgNPs per MB was estimated to be roughly 3200–4600, corresponding to ∼5 pmol of DNA-AgNPs per 1 mg of MBs. For comparison, 1 mg of the MBs can typically bind 500 pmol of single-stranded oligonucleotides, according to the manufacturer. Our loading density was also lower than that of a comparable platinum nano reporter complex (∼280 pmol of ssDNA—PtNP conjugates; 5 nm-sized PtNPs), which can possibly be explained by a higher DNA loading and the larger size of our AgNPs.^49^ In addition, we tested the stability of the final reporter complex by storing it for 1 week in deionized water in the fridge at 4 °C and tested the supernatant after magnetic pull-down for the presence of AgNPs. Since we could not observe AgNPs in the supernatant, we conclude that the reporter complex did not degrade and remained stable for >1 week. A TEM image of the optimized DNA-AgNP-MB reporter complex is shown in Figure S2.

### Magnetic Pull-Down Assay Using UV/Vis Readout

**Figure 4:**
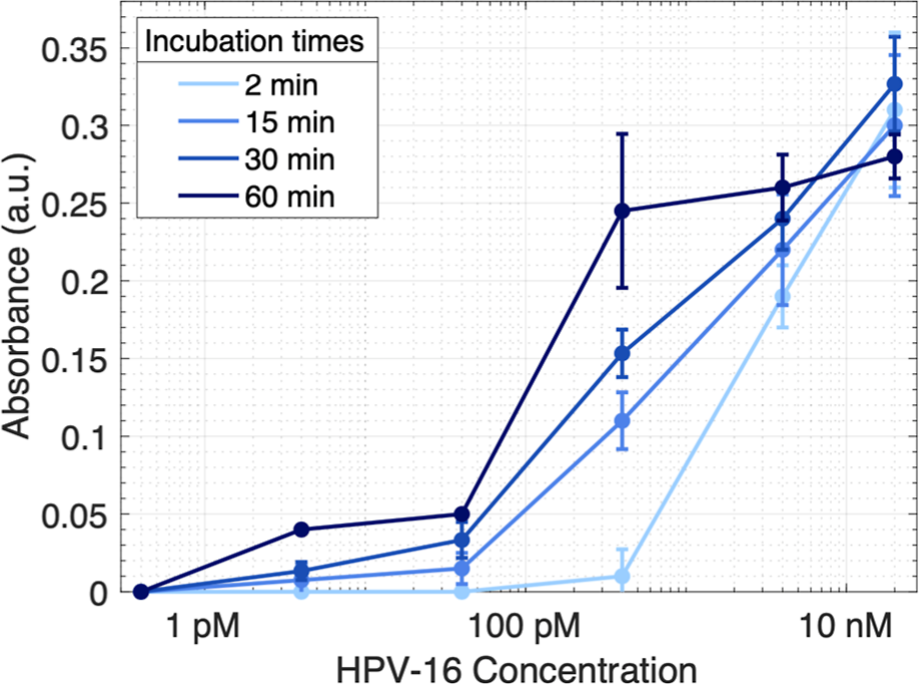
UV/vis readout confirming the concept of the magnetic pull-down assay using the DNA-AgNP-MB reporter complex. The largest dynamic range results from an incubation time of 15 and 30 min. Error bars represent the mean ± standard deviation, where *n* = 3.

First, we established the assay based on UV/vis spectra with the target sequence (HPV-16), without the target, and with a random DNA to confirm the functionality of the optimized DNA-AgNP-MB reporter complex and the selective activation of the RNP complex. As expected, the collateral cleavage activity of Cas12a is only active when the target sequence is used, resulting in the release of DNA-AgNPs (see Figure S3). A minor LSPR peak is visible for the inactive RNP complexes, indicating a very low amount of DNA-AgNPs. This could be due to reporter degradation, a known downside of reporter complexes using ssDNA, or unbound DNA-AgNPs that were not removed by the washing step.^20^ After we confirmed that the reporter complex is functional, we tested different HPV-16 concentrations and measured the supernatant after 2, 15, 30, and 60 min. The kinetics of the enzyme using our reporter complex can be seen in Figure S4. Finally, we determined the incubation time and the dynamic range of the assay using the UV/vis readout by plotting the measured absorbance versus the HPV-16 concentration, as shown in Figure 4. Subsequently, we chose an incubation time of 15 min for further experiments since it enables rapid detection while providing a large dynamic range. Longer incubation times would lead to a lower LOD but conflict with the vision of developing a POC concept.

### Single-Impact Detection of Released DNA-AgNPs

After we validated the assay concept using a standard UV/vis readout, we combined the CRISPR-powered magnetic pull-down with a digital readout concept based on single-impact electrochemistry. For this, we chose AgNPs, which have been abundantly used in direct single-impact electrochemistry due to their ability to be oxidized at moderate potentials. Other nanoparticles, such as AuNPs, require higher oxidation potentials and different electrode materials, e.g., carbon-based electrodes.^50^ Another strategy would be to exploit catalytic properties of metal nanoparticles, as demonstrated for PtNPs. This however generally results in broadened peaks or steps with a lower signal-to-noise ratio (SNR) compared to direct oxidation of nanoparticles.^51−53^ As discussed, there is a trade-off between the size and number of attached ligands to the particle while keeping its redox activity.^54,55^ By choosing a short thiol-modified ssDNA T-spacer (5 nt), we enabled a fast and easy cofunctionalization process, leading to very stable DNA-AgNP conjugates. At the same time, the DNA shell remained thin enough so that the particles could still be oxidized even at low electrode overpotentials (0.3–0.9 V vs Ag/AgCl). Increasing the applied voltage results in an increased mean impact frequency (see Figure S5) due to an extension of the tunneling region and enhanced electrophoretic contributions.^56^ The impact rates also strongly correlate with the DNA loading on the nanoparticle. To investigate this effect, we modified the AgNPs with different molar ratios of activated T-spacers, namely 500:1, 1000:1, and 2000:1. Since the freezing of the particles requires high DNA excess to avoid aggregation, we kept the overall DNA/AgNP ratio constant by adding nonactivated T-spacer to reach a final mixing ratio of 2000:1 for all samples. As expected, the impact rates increased with lower DNA density per particle (see Figure S6a,b). However, at the same time, the AgNP conjugates were less stable and even experienced slight aggregation at a lower mixing ratio of 500:1 (see Figure S6c). In addition, the collateral cleavage activity of Cas12a was highest in 10 mM MgCl_2_, which would cause aggregation of unfunctionalized particles, justifying a higher DNA loading, although compromising the impact yield.

**Figure 5:**
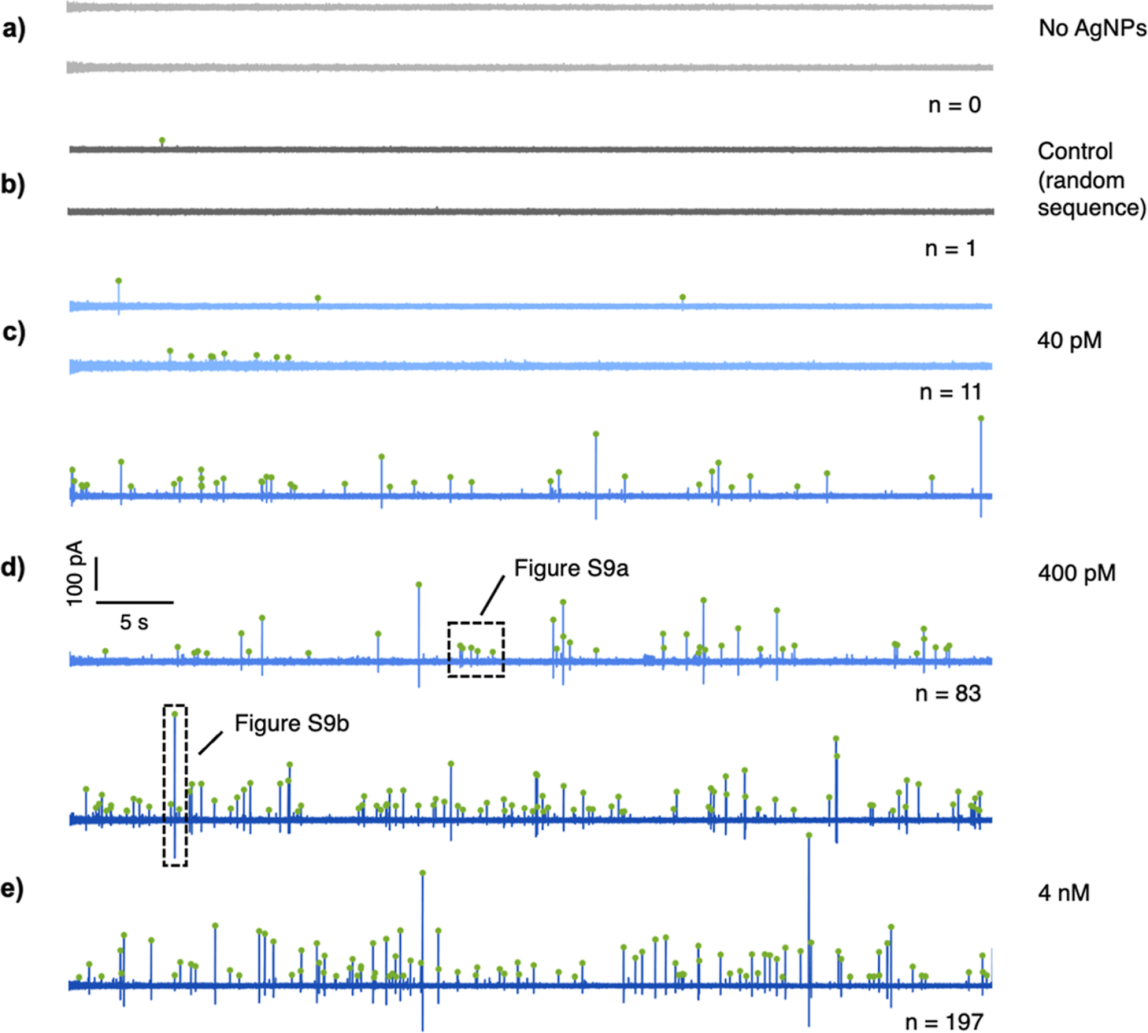
Representative current traces from the single-impact readout of amplification-free digital CRISPR-powered biosensor concept using different target concentrations. (a) Recording in the detection buffer solution shows no AgNP impacts, supporting the conservative current threshold of 25 pA. (b) The control experiment, activating the RNP complex using a random sequence (DNA 1), leads to a single spike, likely due to reporter complex degradation. (c–e) By increasing the target concentration, more AgNP impacts can be counted accordingly. A zoom-in to show the detection threshold and an amplifier-related ringing artifact, indicated in (d,e), is plotted in Figure S7. All recordings were carried out in a total volume of 200 μL containing 200 mM KCl/50 mM KOH, and the microelectrodes were biased to 1 V vs Ag/AgCl.

After verifying the redox activity of the T-spacer particles, we performed the assay using HPV-16 concentrations in the dynamic range visible in Figure 4 (40 pM, 400 pM, and 4 nM) and with a random sequence as negative control. Figure 5 depicts exemplary current traces for different target concentrations and control experiments. The recording in pure detection buffer showed no AgNP impacts (see Figure 5a). Also, the control experiment using a random sequence as an activator resulted in only a single detected peak, most likely due to reporter degradation over time or insufficient washing after coupling the DNA-AgNPs to the MBs, as shown in Figure 5b. We observed increased collision events by increasing the HPV-16 concentration (see Figure 5c–e). Since the Mg^2+^ ions from the reaction buffer reduced the impact efficiency, we used an excess of EDTA (10×) to stop the reaction by chelating the divalent ions (see Figure S7). To facilitate the Mg^2+^ capture, we incubated the released particles in 500 mM NaCl for 10 min and an excess of 10× EDTA. We could not observe any aggregation under these conditions (see Figure S8a,b). The high sodium concentration supports the dissociation of Mg^2+^ ions from the DNA because of competitive binding.^57^ Afterward, the particles were desalted and applied to the MEA chip. As the total number of oxidized particles is very low compared to the number of particles present in the detection solution, the bulk concentration of nanoparticles can be assumed constant throughout the measurement. However, depending on the geometrical arrangement of the electrode array and the reservoir volume, the continuous oxidation of particles during very long measurements can deplete the bulk concentration and must be considered.^58^ To avoid erroneously counting noise or artifacts as AgNP impacts, we chose a conservative detection threshold of 25 pA, as shown in a zoom-in of Figure 5d (see also Figure S9a). Additionally, a magnification from Figure 5e demonstrates the importance of an interpeak distance to avoid misinterpretation of amplifier-related ringing artifacts (see Figure S9b). The noise levels of all impact experiments are given in Figure S10.

**Figure 6:**
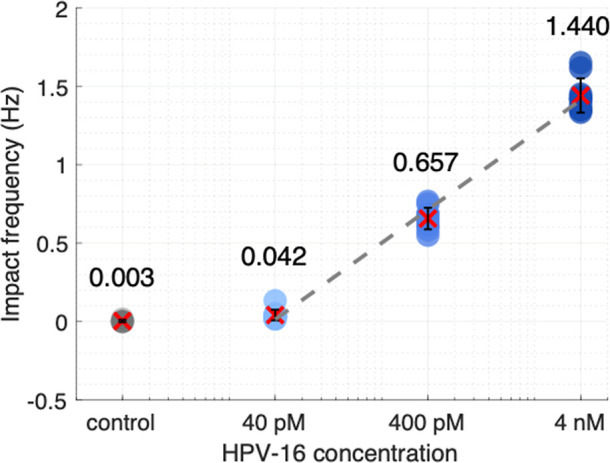
Calibration curve of the amplification-free digital CRISPR-powered biosensor concept. Dots indicate individual channels (*n* = 10); red crosses denote mean values and error bars represent standard deviation.

We analyzed the detected peaks regarding amplitude, duration, and charge transfer to estimate the size distribution (see Figure S11). Interestingly, the released particles tend to transfer less charge when a lower target concentration is present leading to an underestimation of the nanoparticle size. A lower charge transfer is indicative of a partial oxidation of the AgNPs. We hypothesize that as the concentration of activated RNP complexes increased, more of the T-linker was digested, allowing the particles to reach the tunneling region more easily. However, partial oxidation occurs naturally for larger particles (>40 nm) and does not conflict with correlating the overall peak count to the DNA concentration. The stability data of the released particles (see Figure S8b) suggests that possibly not all T-spacers were cleaved by the Cas12a due to steric hindrance, leading to similar stable DNA-AgNP after the cleavage. Finally, we calculated the mean impact frequencies (see Figure 5b–e) and obtained a calibration curve, shown in Figure 6. The LOD can be defined as the target molar concentration required to achieve an end point-based threshold,^59^ given as
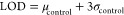
1where μ and σ represent the mean and standard deviation of the negative control with a random sequence. According to this definition, the theoretical LOD of the single impact-based detection shown here was around 25 pM, comparable to the E-CRISPR (50 pM)—a universal electrochemical biosensor based on Cas12a.^17^ It is worth mentioning that we could only calculate a LOD since we counted a single impact for the control experiment. By avoiding AgNPs being in the supernatant, e.g., due to prevention of reporter degradation or more thorough washing steps, the theoretical LOD, given in eq 1, would be infinitesimally low. Clearly, this is not a useful definition for such sensors, as any practical LOD, as well as the sensitivity of the assay, will depend on the incubation and detection times. Nevertheless, it highlights the opportunities that single impact-based digital detection could offer by further improving the detection efficiency of the released particles or scaling up the channel count compared to other CRISPR-based concepts using amplitude-based readouts, such as fluorescence. The sample-to-result time of our implemented sensor concept was ∼50 min, offering a practical benefit to other existing nanoimpact methods that usually take several hours.^30,52,53^ In addition, other nanoimpact methods for DNA detection are often based on catalytically amplified nanoimpacts using catalytical nanoparticles, such as AuNPs and PtNPs, resulting in smaller peak amplitudes. In general, the direct impact of AgNPs leads to higher amplitude peaks, thus providing a higher SNR. Furthermore, the AgNPs in our concept are modified with short T-spacers, providing stability in 500 mM NaCl and indicating their stability and applicability in real samples.

## Conclusion and Outlook

In conclusion, we extended the field of CRISPR-based diagnostics using a digital sensor concept without target amplification based on single-impact electrochemistry for the rapid quantification of HPV-16. We synthesized a highly stable DNA-AgNP-MB reporter complex via a straightforward freezing-directed functionalization, offering a broad application range of potential clinically relevant targets by reprogramming the crRNA, which ultimately could offer multiplexing capabilities. Despite overcoming the magnesium-induced drop in impact frequency of the released DNA-AgNPs by Mg^2+^ replacement and chelation, the detection efficiency remains a primary challenge. Therefore, future efforts should focus on enhancing the mass transfer to the detection electrodes and increasing the oxidation possibility of the particles. This could be addressed, for example, by applying the concept to a paper-based lateral flow setting, microfluidic integration, engineering the electrokinetic transport, or modifying the electrodes with a polysulfide layer.^29,39,60,61^ Furthermore, amplification by multiple collision events using larger nanoparticle events could be a strategy to increase the average impact frequency. The high stability of our DNA-AgNPs indicates their potential use in real samples. However, challenges, such as reporter complex degradation due to the presence of DNases should be addressed by combining our sensing concept with existing strategies, such as heating unextracted diagnostic samples to obliterate nucleases.^62^ We believe that addressing these challenges could help accelerate the development of next-generation digital sensors at the POC for DNA quantification. In addition, the implemented sensor concept could be extended to RNA detection by combining it with the collateral cleavage activity of Cas13a.

## Supporting Information Available

Oligo sequences; material costs of implemented assay; measurement setup; TEM images of DNA-AgNP-MB reporter complex; specificity of RNP activation, Cas12a kinetics, impact frequency in dependence of applied electrode potential; impact frequency in dependence of DNA loading; effect of MgCl_2_ and EDTA on impact rate; stability experiments of released particles; current detection threshold and ringing artifacts; RMS current noise; statistical data of impact experiments (PDF)

## Abbreviations

AgNP: silver nanoparticle
LSPR: localized surface plasmon resonance
MB: magnetic bead
HPV: human papillomavirus
MEA: microelectrode array
CRISPR: clustered regularly interspaced short palindromic repeats
POC: point-of-care
RNP: ribonucleoprotein
