# Supplementary material for: Digital CRISPR-Powered Biosensor Concept without Target Amplification Using Single-Impact Electrochemistry
Source: ACS Sens. 2024 Oct 22;9(11):6197–206. doi: 10.1021/acssensors.4c02060 (PMC11590096; doi:10.1021/acssensors.4c02060)
Supplement: Supplementary file 1 — se4c02060_si_001.pdf [file se4c02060_si_001.pdf]

Supporting Information

Digital CRISPR-Powered Biosensor Concept without Target Amplification Using Single-Impact Electrochemistry

Sebastian Freko[1], Marta Nikić[1], Dirk Mayer[2], Lennart J.K. Weiß[3], Friedrich C. Simmel[3], and Bernhard Wolfrum[1*]

[1] Neuroelectronics, Munich Institute of Biomedical Engineering, Department of Electrical Engineering, School of Computation, Information and Technology, Technical University of Munich, 85748 Garching, Germany

[2] Institute of Biological Information Processing, Bioelectronics (IBI-3), Forschungszentrum Jülich, 52425 Jülich, Germany

[3]Department of Bioscience, TUM School of Natural Sciences, Technical University of Munich, 85748 Garching, Germany

[*] Corresponding author: Bernhard Wolfrum, bernhard.wolfrum@tum.de

I. Oligonucleotide Sequences

Table S1. *Sequences of oligonucleotides used in this work.*

Name	Sequences (5'-3')
**T-spacer**	SH-TTTTT
**Biotin/T-spacer**	SH-T(x100)-Biotin
**Random sequence**	TCA CAG ATG CGT AAA AAA AAA
**TS HPV 16**	CAA ATA TGT CAT TAT GTG CTG CCA TAT CTA CTT CAG AAA CTA CAT ATA A
**Lb-crRNA-HPV-16**	UAA UUU CUA CUA AGU GUA GAU TGA AGT AGA TAT GGC AGC AC

II. Material Costs of Implemented Assay

Table S2. Summarized material costs of the implemented sensor concept.

Name	Costs/Assay
AgNPs	0.40 €
T-spacer	1.50 €
Biotin/T-spacer	0.36 €
LbCas12a	0.04 €
Lb-crRNA-HPV-16	< 0.01 €
Magnetic beads	1.80 €
**Total per assay**	**4.10 €**

III. Measurement Setup for Single-Impact Recordings

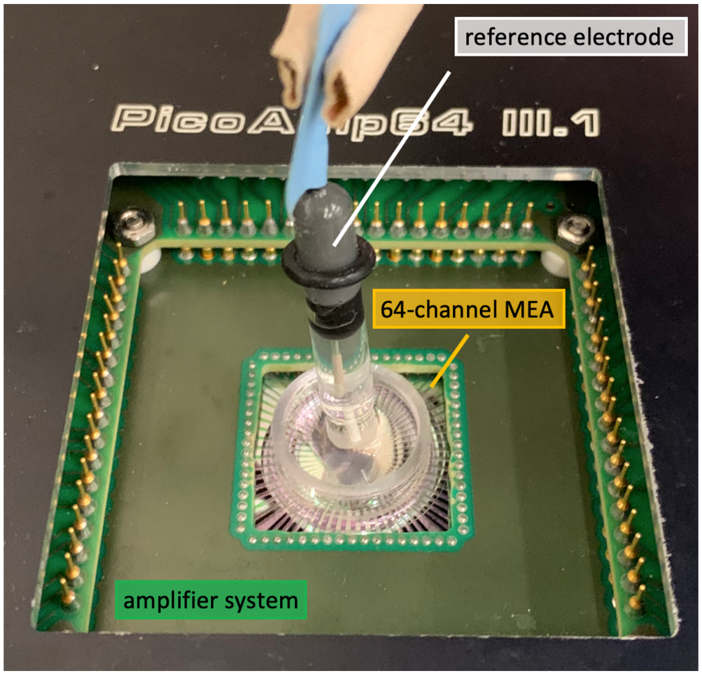

Figure S1. *The detection experiments were carried out in a 2-electrode configuration using a custom-built 64-channel amplifier system.*

IV. TEM Image of Optimized DNA-AgNP-MB Reporter Complex

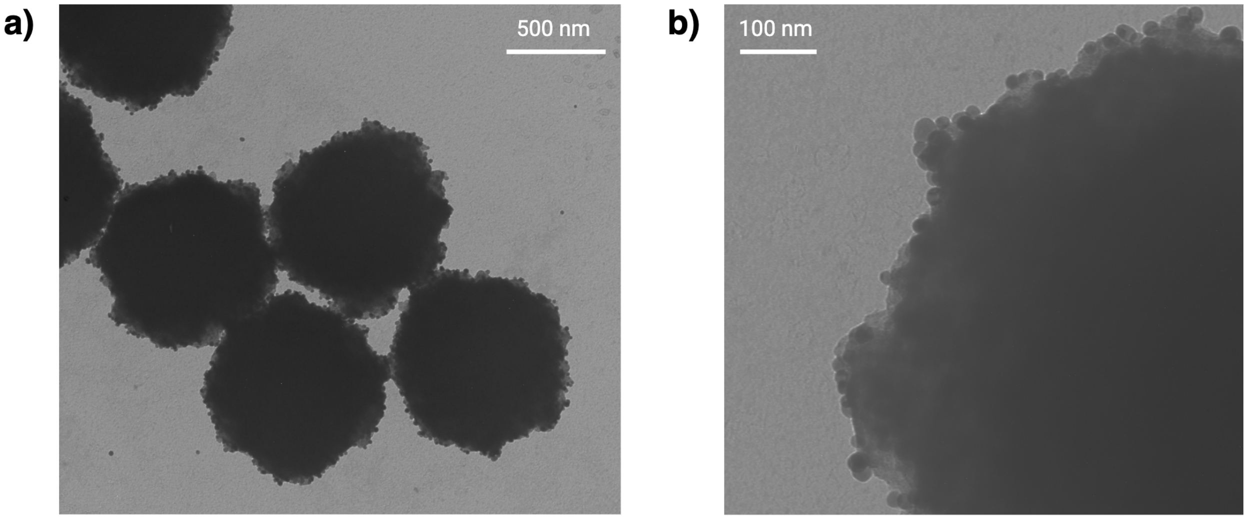

Figure S2. *Representative TEM image of the optimized DNA-AgNP-MB reporter complex. a) Cluster of MBs cross-linked by AgNPs carrying more than one T-linker. b) Close-up of immobilized AgNPs on MB surface. Images were obtained at 100 kV.*

V. Specificity of RNP Activation

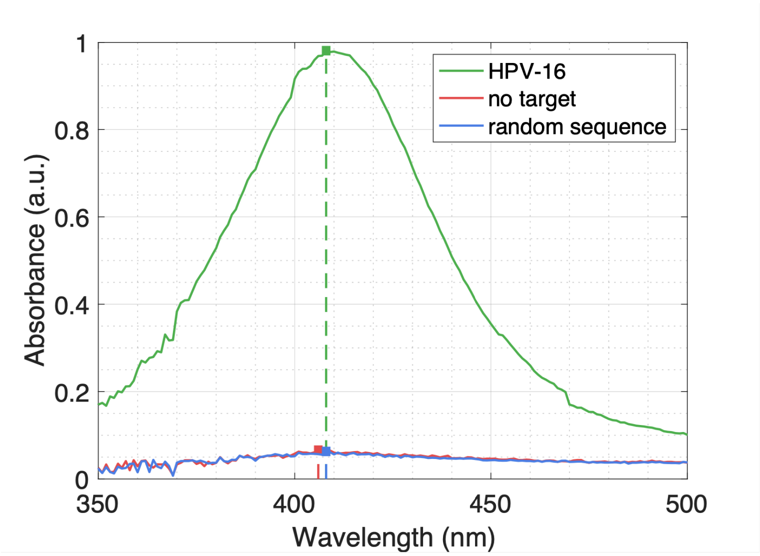

Figure S3. *The RNP complexes were mixed with the target sequence (HPV-16), a random sequence, and no DNA was added. As expected, only the target sequence activated the RNP complex, leading to reporter cleavage and particle release, shown by the prominent peak of the supernatant. The samples with no target and the random sequence show only a minimal LSPR peak at the expected wavelength, likely due to reporter degradation over time or insufficient washing after coupling the DNA-AgNPs to the MBs. The exact experiment conditions are described in the methods section of the manuscript.*

VI. Kinetics of Cas12a Using DNA-AgNP-MB Reporter Complex

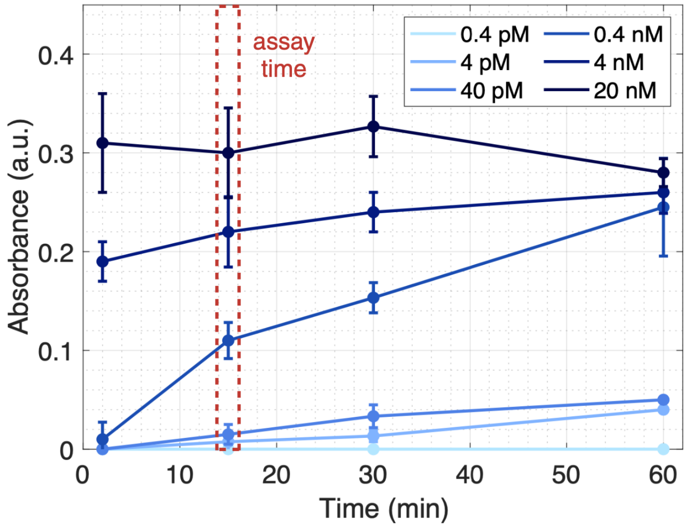

Figure S4. *The reaction kinetics of the Cas12a enzyme using the DNA-AgNP-MB reporter complex. This Figure shows the same data as Figure 4 in the manuscript but depicts the absorbance as a function of incubation time. Error bars represent the mean ± standard deviation, where n = 3.*

VII. AgNP Impacts at Different Applied Electrode Potentials

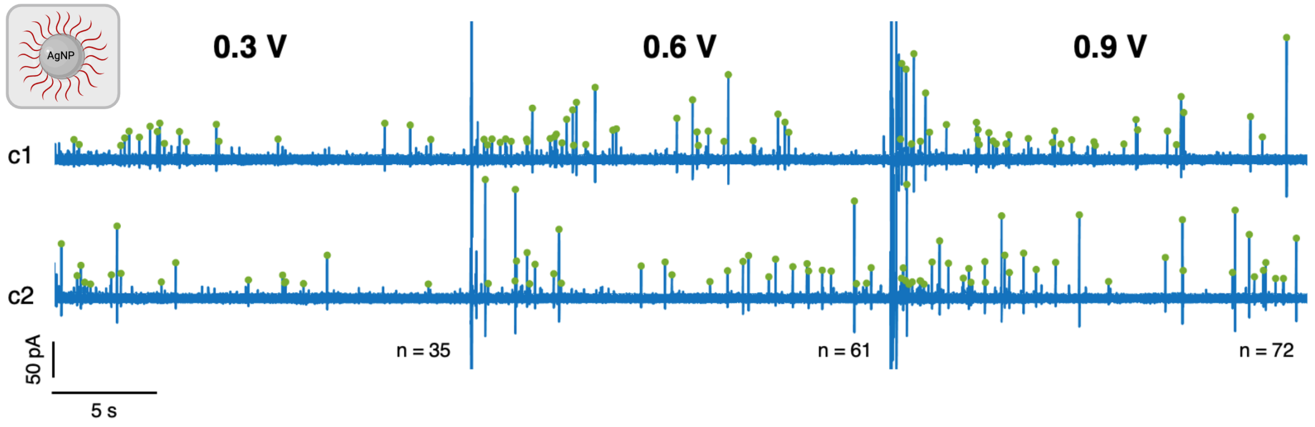

Figure S5. *Number of particle oxidation events of T-spacer-AgNPs in dependence on the applied potential vs. Ag/AgCl. T-spacer-AgNPs can be (partially) oxidized. The detection efficiency increases with higher potentials at the electrodes. The experiment was carried out in a total volume of 200 µL containing 200 mM KCl / 50 mM KOH.*

VIII. Impact Frequency in Dependence of DNA Loading on AgNPs

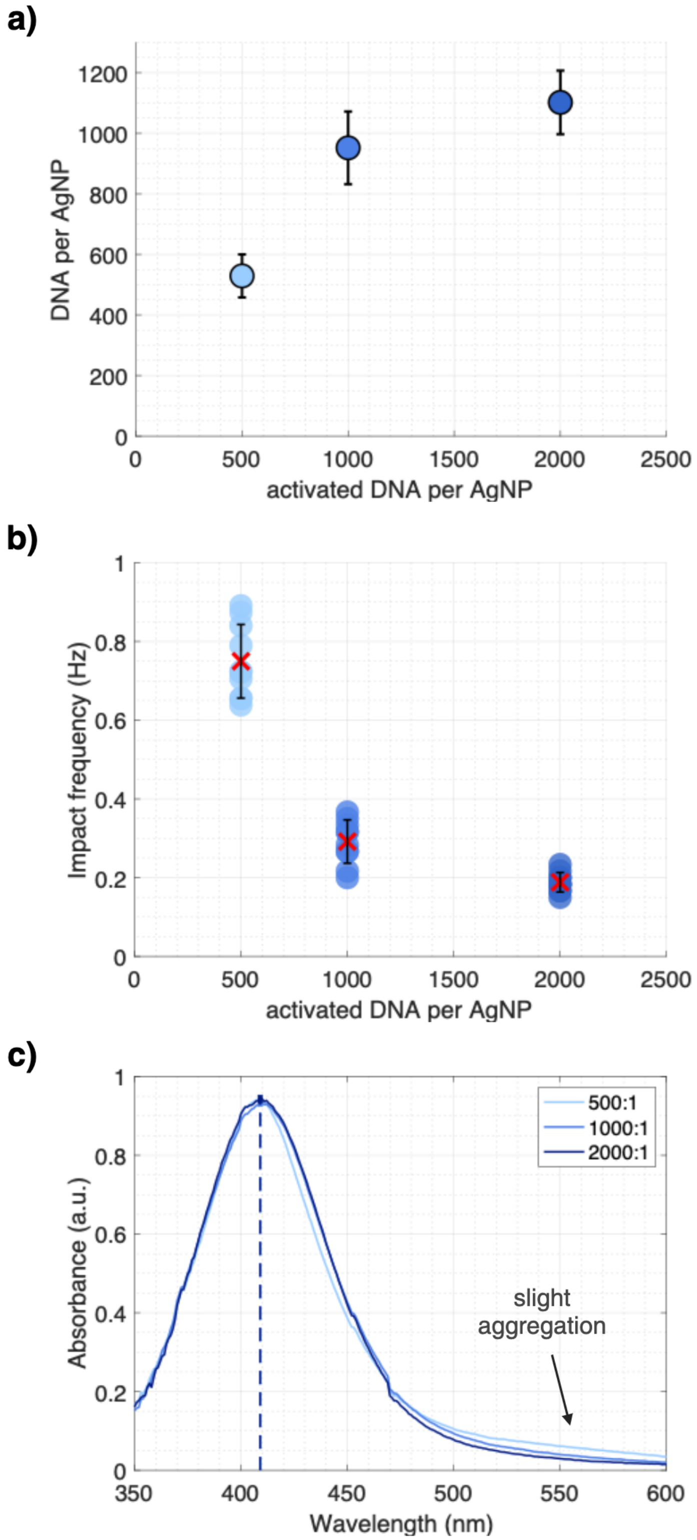

Figure S6. *Influence of the DNA loading density on the impact rate. AgNPs were functionalized using a molar ratio of 2000:1, but only different fractions of T-spacers were activated using TECP (500:1, 1000:1, and 2000:1). a) Plot of the number of bound DNA strands per AgNP vs the total number of activated DNA strands per AgNP before the reaction. DNA loading increased with the activated DNA added per particle before saturation. Error bars represent the mean ± standard deviation, where n = 3. b) Accordingly, the impact rate decreases inversely proportional to the DNA loading. Dots indicate individual channels (n = 10); red crosses denote mean values and error bars represent standard deviation. c) Slight aggregation occurred during the freezing-directed functionalization process due to fewer activated T-spacers present.*

IX. Direct Effect of $MgCl_2$ and EDTA on Impact Efficiency

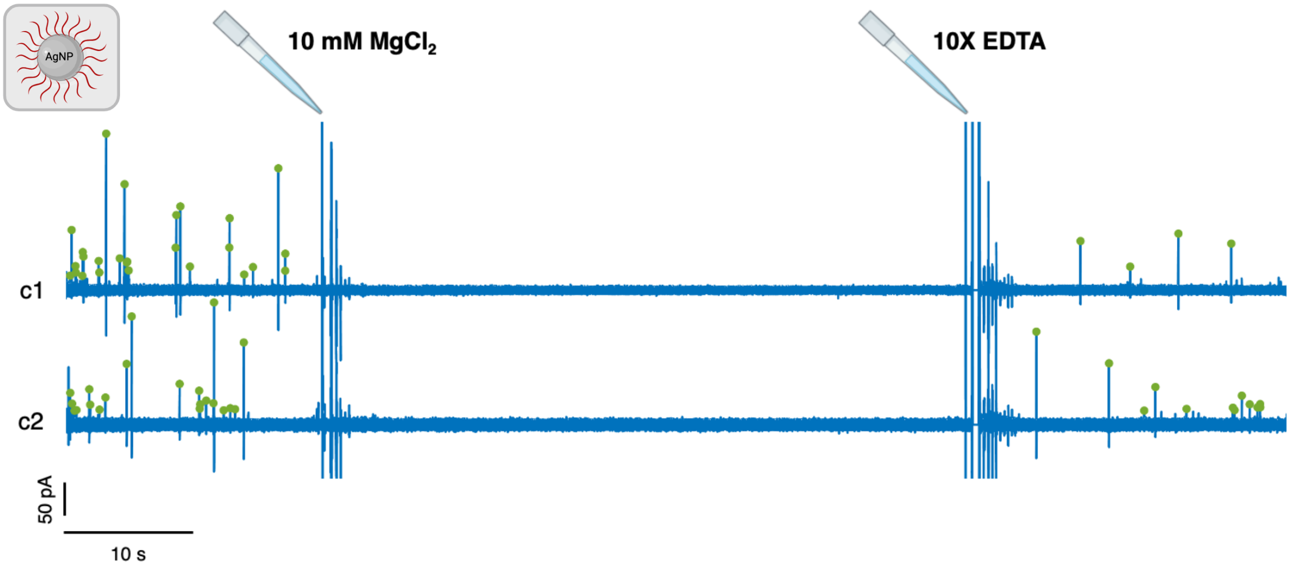

Figure S7. Direct effect of $MgCl_2$ on the detection ability. The recording started in the detection buffer (200 mM KCl / 50 mM KOH). After adding 10 mM $MgCl_2$ (final concentration), AgNP impacts diminished immediately. We assume that the Mg^{2+} ions bind to the negatively charged backbone of the DNA. By adding 10X excess EDTA, the impact rate could be partially recovered, demonstrating the importance of neutralizing positively charged divalent magnesium ions. To prevent pipetting-related artifacts from being incorrectly counted as impacts when adding the chemicals, an exclusion time of 6 s was selected. The experiment was carried out in a total volume of 200 μL containing 200 mM KCl / 50 mM KOH, and the microelectrodes were biased to 0.9 V vs. Ag/AgCl.

X. Stability Experiments of Released Particles

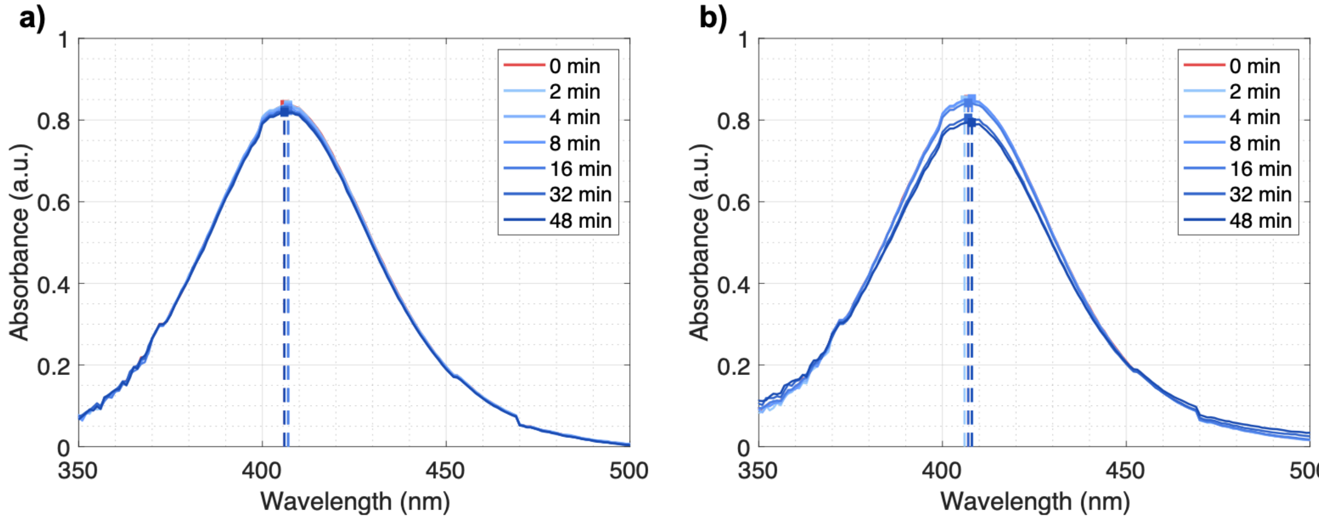

Figure S8. Stability test of released particles in a) 10X EDTA and b) 10X EDTA + 500 mM NaCl. a) EDTA excess does not influence the UV/Vis spectra after 48 min. b) 500 mM NaCl leads to a decrease in amplitude, indicating slight aggregation for t > 16 min. The particles were released using a target concentration of 4 nM and an incubation time of 15 min.

XI. Current Detection Threshold and Ringing Artifacts

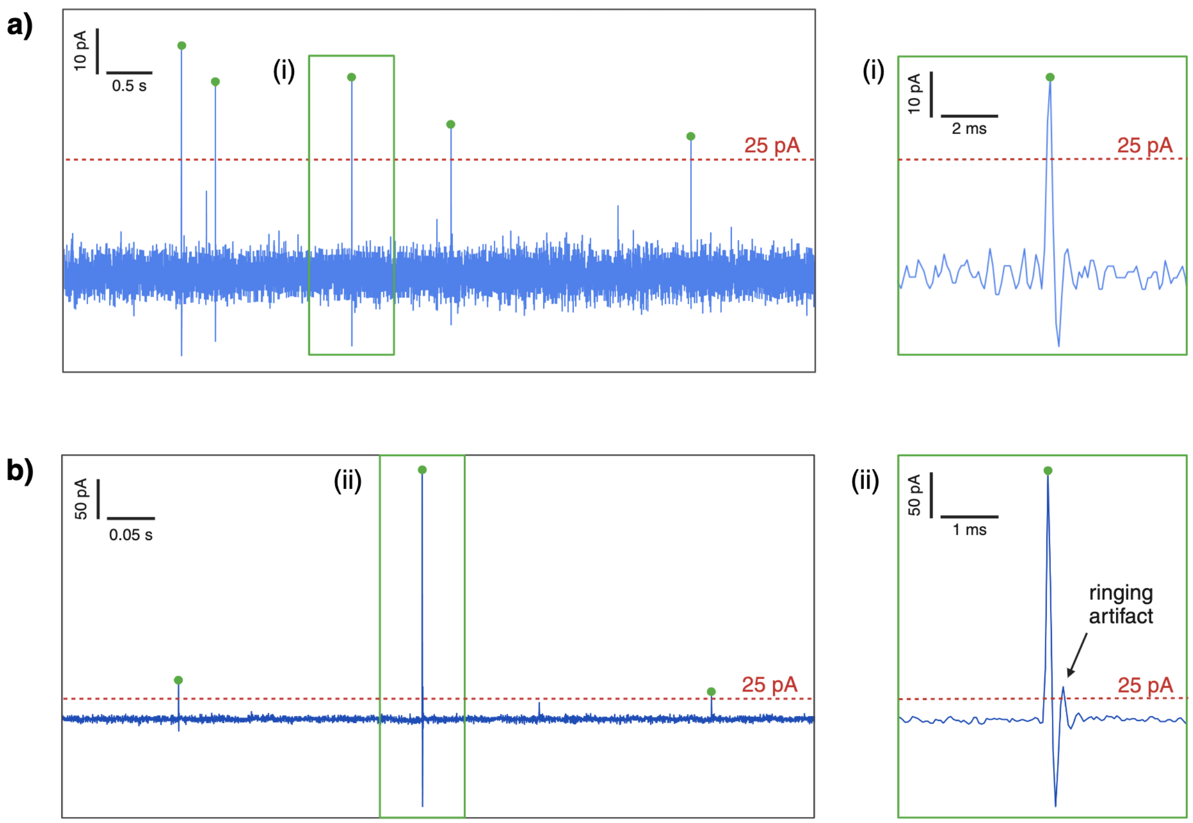

Figure S9. *Zoom-in on current traces of impact experiments from the calibration curve (Figure 4), showing a) the detection threshold of 25 pM and b) an amplifier-related ringing artifact after initial charge injection of a high-amplitude AgNP impact. The conservatively selected current threshold and a minimum inter-peak distance of 10 ms prevent misclassification of peaks.*

XII. RMS Current Noise

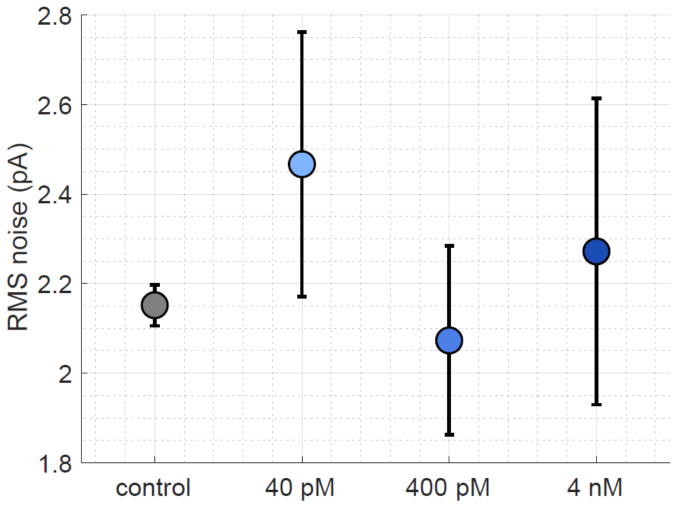

Figure S10. *RMS current noise of impact experiments from the calibration curve. Error bars represent the mean ± standard deviation, where n = 10.*

XIII. Statistical Data of Impact Detection of Released Particles

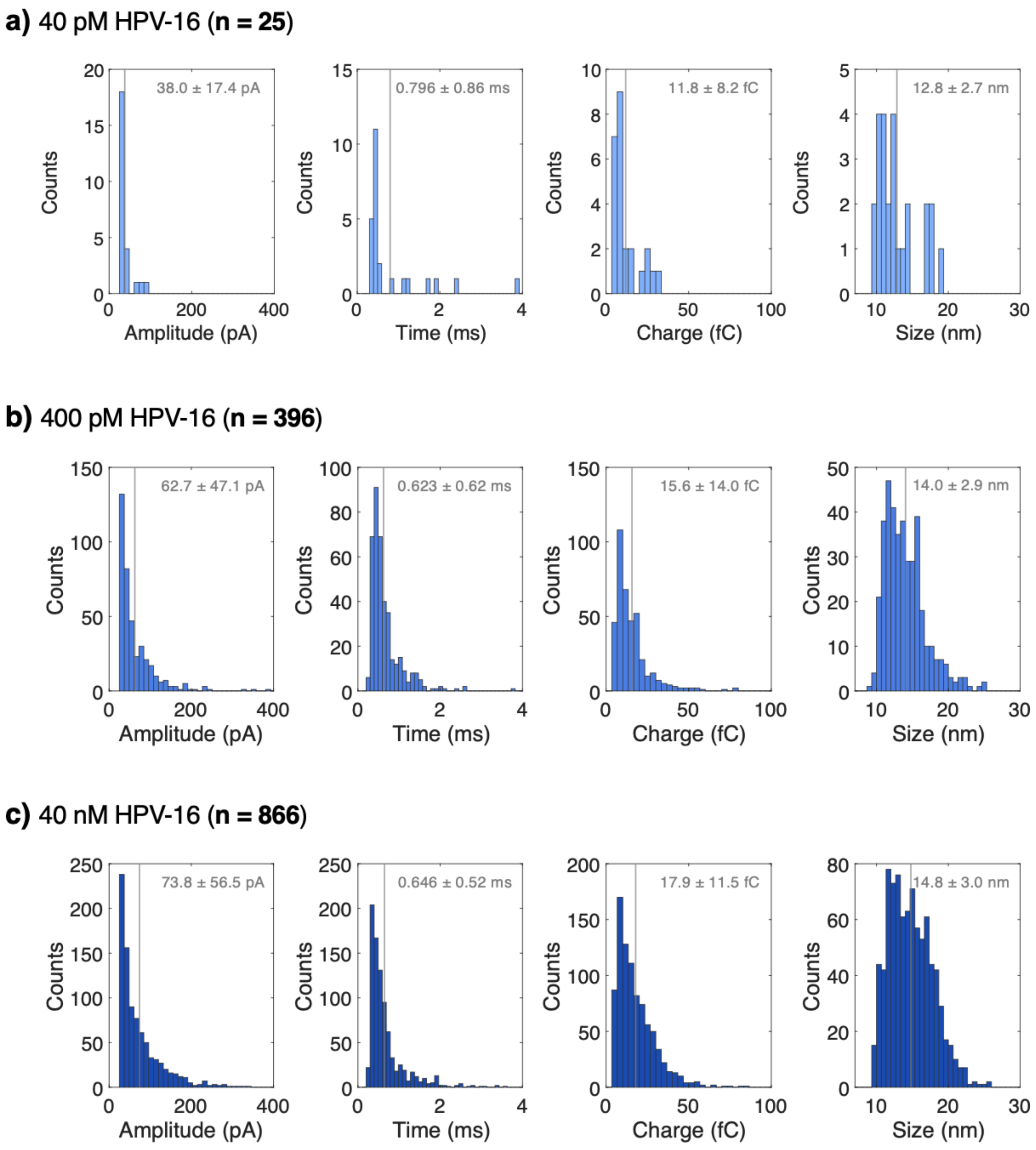

Figure S11. *Statistical peak analysis of recordings from the calibration curve (Figure 5), including amplitude, duration, charge, and size distributions. a-c) The released particles tend to be oxidized more completely when a higher target concentration is present, which is indicated by the increased transferred charge per particle. This is also reflected in the larger estimated particle size. Mean values and corresponding standard deviations are shown in grey.*
